# Impact of environmental temperature on production traits in pigs

**DOI:** 10.1038/s41598-020-58981-w

**Published:** 2020-02-07

**Authors:** Wendy M. Rauw, Eduardo de Mercado de la Peña, Luis Gomez-Raya, Luis Alberto García Cortés, Juan José Ciruelos, Emilio Gómez Izquierdo

**Affiliations:** 1 Departamento de Mejora Genética Animal, Instituto Nacional de Investigación y Tecnología Agraria y Alimentaria, Crta de la Coruña km 7.5, 28040 Madrid, Spain; 2 Centro de Pruebas de Porcino, Instituto Tecnológico Agrario Junta de Castilla y León, Crta Riaza-Toro S/N, 40353, Hontalbilla, Spain

**Keywords:** Animal biotechnology, Zoology

## Abstract

There is an urgent need to identify the effects of temperature on production traits. This study aimed to determine the impact of pig production in three environments (T_°Cgrowing-°Cfattening-°Cfinishing_ = T_24-24-21_, T_19-19-19_, and T_23-17-15_) on growth curve parameters, body weight gain (DBWG), feed intake (DFI), and feed efficiency during the growing, fattening and finishing stages, and on carcass yield of primal cuts (ham, shoulder, and loin) in 158 Duroc × Iberian pigs. Maturation rate was higher in T_23-17-15_ than in T_19-19-19_ (P < 0.001). Pigs in T_23-17-15_ reached a lower mature body weight (P < 0.05). During the growing stage, pigs in T_23-17-15_ had higher DFI than those in T_24-24-21_ and T_19-19-19_ (P < 0.05); during the fattening stage, DFI was lowest in T_24-24-21_ (P < 0.001). In the growing stage, pigs had highest DBWG in the warmest environments (T_24-24-21_ and T_23-17-15_) and lowest in the coldest environment (T_19-19-19_; P < 0.001). Feed efficiency was highest in warmer environments (P < 0.01). Temperature T_24-24-21_ favored loin yield, T_19-19-19_ favored ham yield, and T_23-17-15_ favored shoulder yield (P < 0.01). The results imply a favorable effect of temperature on feed efficiency, however, possible negative implications for animal health and welfare should be considered.

## Introduction

Seasonal variation in pig production has been implicated in variation in productive and reproductive performance through direct and indirect effects of temperature on physiological responses, including intestinal integrity, endocrine signaling, adipose mobilization, and insulin regulation^1,2^. Heat stress in pig production results in industry losses resulting from slower growth rates, inconsistent market weights, altered carcass traits, infertility, increased health care costs and mortality^3^. For example, results by Rinaldo *et al*.^4^ indicated that, in the tropics, growth performance varied with the season and that during the warm season, feed intake was a major limiting factor to body weight gain. Collin *et al*.^5^ observed a clear decrease in voluntary feed intake (45 g/d) and in body weight gain (37 g/d) in group-housed young pigs between 23 and 33 °C. A meta-analysis by Renaudeau *et al*.^6^ showed that high temperatures have a curvilinear undesirable effect on voluntary feed intake and body weight gain, and that this effect is more pronounced in heavier pigs. The negative implication of heat stress will likely become more of an issue if the frequency of severe hot weather increases as predicted;^7^ also in Spain, increased summer temperatures resulting from climate change are expected to jeopardize profitability of livestock production because of reduced production and increased levels of stress^8^. Therefore, there is an urgent need to identify the effects of temperature on production traits.

In Spain, in a production system called ‘montanera’, Iberian pigs roam the Mediterranean forest called the “dehesa”, which are woodlands of evergreen oaks^9^. The most valuable meat product is the dry-cured ham which has a very distinctive flavor. Iberian pig meat can show an intramuscular fat content of up to nearly 20%, which is considerably higher than that described in pork from commercial white breeds^10^. However, because of limited land availability and low production levels, Iberian pigs are regularly crossed with Duroc producing either 50% or 75% Iberian fattening pigs. In the first trimester of 2015, this constituted 70% and 11% of the total Spanish Iberian pig production, respectively; 44% of the total production constituted Duroc × Iberian pigs fed intensively on concentrate^11^. Although the cross with Duroc improves growth rate and muscle marbling, intensive production on concentrate feeding results in a reduction of meat quality^12^.

The objective of the present study was to determine the impact of pig production in three seasons at different temperatures on growth curve parameters, body weight gain, feed intake, feed efficiency, and carcass yield of primal cuts (ham, shoulder, and loin). The second objective was to determine the relationship between these traits in the Duroc × Iberian breed. The temperatures hovered around 23 °C in a summer replicate, around 19 °C in a winter replicate with temperature control, and between 25–15 °C in an autumn replicate with temperature control. Whereas literature on trends in weight gain and feed intake in Duroc × Iberian pigs is scarce, to our knowledge this is the first study describing feed efficiency in this cross. To our knowledge it is also one of few studies describing trends in weight gain, feed intake, and feed efficiency in pigs up to an advanced physiological age.

## Methods

### Animals and management

All procedures followed the Spanish policy for the protection of animals used in research and other scientific purposes RD53/2013. The project was approved by the ITACyL Ethics Committee on Animal Experimentation, reference number 2018/37/CEEA. A total of 158 Duroc x Iberian crossbred barrows in three replicates (53 in T_24-24-21_, 55 in T_19-19-19_, and 50 in T_23-17-15_, respectively) were used in this experiment. Pigs originated from a production farm belonging to Grupo Copese, S.A. located in Coca (Segovia, Spain) and were individually marked before the start of the experiment. The trial was conducted at the Swine Research Center of the Agricultural Technological Institute of Castilla and Leon (ITACyL) in Hontalbilla, Segovia. Within temperature group, animals were housed individually in one of 14 pens in one of 4 rooms. Each pen was equipped with a stainless-steel feeder and a nipple drinker. Feed and water were provided *ad libitum* during the entire experiment.

In T_24-24-21_, animals were evaluated between June 14 and October 16, 2017 (a total of 124 days); in T_19-19-19_, animals were evaluated between January 3 and June 6, 2018 (a total of 154 days); in T_23-17-15_ animals were evaluated between August 7 and December 19, 2018 (a total of 134 days). Age at the start of the evaluation was unknown for any of the 158 individuals. Within temperature group, in function of the average body weight, three diets formulated by Grupo Copese, S.A. were fed during the growing-finishing stage: a growth formula, a fattening formula, and a finishing formula (Table 1). Animals grew from an average body weight of 40.6 (SD 3.5) kg to 158.1 (SD 11.1) kg in T_24-24-21_, from 35.5 (SD 5.1) kg to 170.5 (SD 15.4) kg in T_19-19-19_, and from 32.4 (SD 6.1) kg to 155.6 (SD 13.1) kg in T_23-17-15_. Within temperature group, pigs were weighed and feed intake (FI) was recorded every 6 to 8 days: 19 times in T_24-24-21_, 23 times in T_19-19-19_, and 20 times in T_23-17-15_. A measured amount of feed was added daily in excess and leftover feed was weighed at the end of each period to calculate the total consumption for each period.

**Table 1:** Diet composition in the growing, fattening, and finishing stage as specified by the producer.

Item	Growing	Fattening	Finishing
Net energy (kcal/kg)	2258	2498	2420
Crude protein, %	13.7	12.5^a^	12.75
Lysine, %	0.76	0.68	0.55
Methionine, %	0.27	0.23	0.23
Crude fat, %	4.1	7.0^b^	5.1
Crude fiber, %	4.6	4.2	4.5
Crude ash, %	6	4.6	4.6
Calcium, %	0.77	0.48	0.51
Phosphorus, %	0.48	0.42	0.44
Sodium, %	0.24	0.23	0.25

^a^1% higher in T_24-24-21_; ^b^0.5% higher in T_24-24-21_.

### Growth curve parameters

Following Rauw *et al*.^13^, modified Parks’^14^ curves were fitted with the non-linear function in SAS (proc NLIN) to individual data of body weight against cumulative feed intake:

1 $${{\rm{BW}}}_{{\rm{t}}}={\rm{A}}\,(1\,\mbox{--}\,{e}^{-{\rm{B}}({{\rm{CFI}}}_{{\rm{t}}}+{{\rm{FI}}}_{0})}),$$

where BW_t_ = body weight of the individual (kg) at day t (days on trial); CFI_t_ = cumulative feed intake (kg) at day t (days on trial; on day 1 CFI = 0); A = mature (adult, asymptotic) body weight (kg); B = rate of maturation of body weight with respect to feed intake (per kg); FI_0_ = a translation of Eq. (1) along the X axis to complete the description of growth (kg). A, B and FI_0_ are parameters to be estimated; only parameters A and B will be discussed. The modification of the Parks’ curve involves the inclusion of FI_0_ to avoid fixing the curve through any point^15^. Parameter estimates were only considered for individuals to which the convergence criterion was met.

Subsequently, a linear function by Parks^14^ was used that related individual cumulative feed intake to day on trial to estimate daily feed intake in mature animals (MFI; kg/d); to ensure that cumulative intake increased linearly, MFI was calculated for measurements during the fattening and finishing stages only.

2 $${{\rm{CFI}}}_{{\rm{t}}}=\,{\rm{Int}}\,+({\rm{MFI}}\times {\rm{Day}}),$$

where CFI_t_ = cumulative feed intake of the animal (kg) at day t, Int = intercept, MFI = mature (maximum daily) feed intake (kg/d), and Day = day on trial (after 48 d). Int and MFI are parameters to be estimated; only MFI will be discussed.

### Average daily body weight gain, feed intake, and mature feed intake

**Figure 1:**
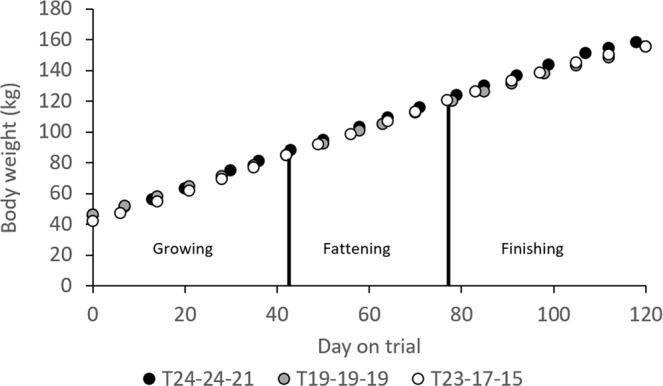
Body weight as a function of day on trial, in three temperature groups (T_24-24-21_, T_19-19-19_, T_23-17-15_).

Because pigs in different temperature groups entered the experiment at different body weights and age was unknown, average daily body weight gain (DBWG; kg/d) and average daily feed intake (DFI; kg/d) were calculated for 6 consecutive periods (approximately one week each) between an average body weight of 45.7 to 88.2 kg, 46.2 to 84.8 kg, and 42.3 to 85.0 kg, in T_24-24-21_, T_19-19-19_, T_23-17-15_, respectively (growing stage), for 5 consecutive periods up to an average body weight of 124.2, 120.2, and 120.6 kg in T_24-24-21_, T_19-19-19_, T_23-17-15_, respectively (fattening stage), and for 6 consecutive periods up to an average body weight of 158.1, 155.4, and 155.6 kg in T_24-24-21_, T_19-19-19_, T_23-17-15_, respectively (finishing stage) (Fig. 1). The average BW of pigs at the start of the growing stage was similar in T_24-24-21_, T_19-19-19_, but a few kilos lower in T_23-17-15_ (P < 0.01). The total time spanning the 17 periods was 118 (20-06 to 16-10-2017), 120 (17-01 to 17-05-2018), and 120 days (21-08 to 19-12-2018) for T_24-24-21_, T_19-19-19_, T_23-17-15_, respectively. The feed formulation during the growing, fattening, and finishing stages corresponded to the growing, fattening, and finishing diets (Table 1).

**Figure 2:**
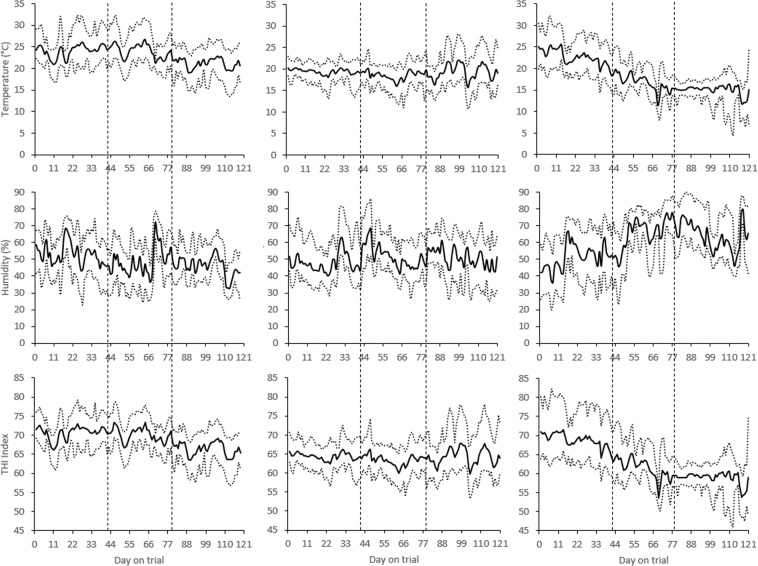
Maximum, average, and minimum temperatures, humidity, and temperature-humidity-index (THI) values in T_24-24-21_ (left panels), T_19-19-19_ (middle panels), and T_23-17-15_ (right panels). Vertical dotted lines indicate the successive stages of growing, fattening, and finishing.

The average, minimum and maximum temperature, humidity, and temperature-humidity-index (THI) values for the four rooms for each of the three temperature groups is given in Fig. 2. THI was estimated as THI = 0.8 × T + RH × (T-14.4) + 46.4; T = temperature in °C, and RH = relative humidity expressed as a proportion. In T_24-24-21_, temperatures hovered around 24 °C in the growing and fattening stages, and a few degrees lower in the finishing stage (overall mean of 24.1 [s.d. 2.67], 24.0, [s.d. 2.85], and 21.1 [s.d. 2.62] °C, in the growing, fattening, and finishing stage, respectively); in T_19-19-19_, temperatures hovered around 19 °C in all stages (overall mean of 19.4 [s.d. 1.33], 18.5, [s.d. 1.82], and 19.3 [s.d. 2.87] °C, in the growing, fattening, and finishing stage, respectively); in T_23-17-15_, temperatures decreased from around 25 to 20 °C in the growing stage, decreased from about 20 to 15 °C in the fattening stage, and hovered around 15 °C in the finishing stage (overall mean of 22.6 [s.d. 3.28], 16.6, [s.d. 2.82], and 14.7 [s.d. 2.30] °C, in the growing, fattening, and finishing stage, respectively). Overall average humidity was 49.2 (s.d. 9.31), 50.1 (s.d. 8.82), and 59.9 (s.d. 13.8) % in T_24-24-21_, T_19-19-19_, T_23-17-15_), respectively (Fig. 2).

### Feed efficiency

Two methods were used to evaluate feed efficiency: (1) feed conversion efficiency (FCE), and (2) residual feed intake (RFI). Higher FCE and lower RFI imply a higher feed efficiency. Feed conversion efficiency was calculated individually as DBWG/DFI for each of the 17 periods, and for the growing, fattening, and finishing stages. Residual feed intake is defined as the difference between the actual DFI and that predicted from a linear multiple regression of DFI on metabolic body weight (BW^0.75^), DBWG, and, if available, a measure of body composition, and is therefore phenotypically independent of growth rate and size^16^. Following Rauw *et al*.^17^, the equation used to estimate RFI was based on the following multiple linear regression of DFI on BW^0.75^, DBWG, and BFT (measured at slaughter) including all measurements on all 158 individuals in all 17 periods in T_24-24-21_, T_19-19-19_, and T_23-17-15_:

3 $${{\rm{DFI}}}_{{\rm{i}}}={{\rm{b}}}_{0}+{(b}_{1}\times {{{\rm{BW}}}_{{\rm{i}}}}^{0.75})+{(b}_{2}\times {{\rm{DBWG}}}_{{\rm{i}}})+{(b}_{3}\times {{\rm{BFT}}}_{{\rm{i}}})+{{\rm{e}}}_{{\rm{i}}},$$

where DFI_i_ = average daily feed intake of individual i (kg/d), BW_i_^0.75^ = average metabolic body weight of individual i (kg^0.75^), DBWG_i_ = average daily body weight gain of individual i (kg/d), BFT_i_ = backfat thickness of individual i at slaughter (mm), b_0_ is the population intercept, b_1_, b_2_, and b_3_ are the partial regression coefficients representing average maintenance requirements per kg metabolic body weight, average feed requirements for DBWG, and average feed requirements related to variation in BFT at slaughter, respectively; e_i_ is the error term, which represents the RFI of individual i, in kg/d. Metabolic BW was estimated as the average metabolic BW for the 17 periods. Subsequently, RFI was calculated for each individual for each of the 17 periods, and for the growing, fattening, and finishing stages as:

4 $${{\rm{RFI}}}_{{\rm{ip}}}={{\rm{DFI}}}_{{\rm{ip}}}-\{{\hat{{\rm{b}}}}_{0}+({\hat{{\rm{b}}}}_{1}\times {{{\rm{BW}}}_{{\rm{ip}}}}^{0.75})+({\hat{{\rm{b}}}}_{2}\times {{\rm{DBWG}}}_{{\rm{ip}}})+({\hat{{\rm{b}}}}_{3}\times {{\rm{BFT}}}_{{\rm{i}}})\},$$

where DFI_ip_ = average daily feed intake of individual i in period or stage p (kg/d), BW_ip_^0.75^ = average metabolic body weight of individual i in period or stage p (kg^0.75^), DBWG_i_ = average daily body weight gain of individual i in period or stage p (kg/d), and BFT_i_ = as in model (3).

### Slaughter yield

One day after the last BW measurement (BWsl), all animals were slaughtered at the processing plant of Comercial Pecuaria Segoviana S.L. (Grupo Copese, S.A.) in Coca, Segovia. Pigs were stunned with CO_2_, exsanguinated, scaled, and eviscerated according to standard commercial procedures, and split down the center of the vertebral column. Hot carcass weight (HCW) was taken (with head); dressing percentage (Dressing%) was calculated as a percentage of BWsl. Entire loins (*Longissimus dorsi*) were removed from the vertebral column, which is common practice in the processing of slaughtered Iberian pigs and their crosses. Backfat depth (BFT) was measured between the third- and fourth-last ribs on the midline of the carcass (including the skin). Subsequently, one loin, ham, and shoulder were trimmed of external fat and weighed (LoinW, HamW, and ShoulderW, respectively). Slaughter yields were furthermore expressed as a percentage of HCW (Loin%, Ham%, and Shoulder%, respectively) and ShoulderW was expressed as a proportion of HamW (ShoulderW:HamW)

### Statistical analyses

The SAS program (SAS Institute, Cary, USA) was used for the statistical analyses of all traits. Individual repeated measurements of DBWG, DFI, FCE, and RFI during the growing, fattening, and finishing stages were analyzed with the mixed linear model procedure (proc mixed):

5 $${{\rm{Y}}}_{{\rm{ijk}}}={\rm{\mu }}+{{\rm{GrowthStage}}}_{{\rm{i}}}+{{\rm{Temperature}}}_{{\rm{j}}}+{(\text{GrowthStage}\times {\rm{Temperature}})}_{{\rm{ij}}}+{{\rm{e}}}_{{\rm{ijk}}},$$

where Y_ijk_ = the phenotype measured on animal k, GrowthStage_i_ = effect of growth stage i (fixed effect; growing, fattening, finishing), Temperature_j_ = effect of temperature group j (fixed effect; T_24-24-21_, T_19-19-19_, T_23-17-15_), and (GrowthStage × Temperature)_ij_ = the interaction effect of growth stage i × temperature group j; e_ijk_∼NID(0, δ^2^e). Initially, the effect of room was also included in the model, but since this was not significant it was removed. Growth stage was identified as the repeated effect in the model for each individual. The following variance-covariance structures for repeated measures were evaluated to describe individual observations on a trait by trait basis: Homogeneous Autoregressive(1) (AR(1)), Heterogeneous Autoregressive(1) (ARH(1)), Compound Symmetry (CS), Toeplitz (TOEP), and Unstructured (UN). The first two models also included the random effect of the individual. Model choice was based on evaluation of fit statistics [the (corrected) Akaike’s information criterion and the Sawa Bayesian information criterion]. Based on the fit statistics, DBWG, DFI, and RFI were analyzed with the ARH(1) model; FCE was analyzed with the CS model.

Slaughter traits were analyzed with the general linear model (proc glm):

6 $${{\rm{Y}}}_{{\rm{ijk}}}={\rm{\mu }}+{{\rm{Temperature}}}_{{\rm{i}}}+{{\rm{HCW}}}_{{\rm{j}}}+{{\rm{e}}}_{{\rm{ijk}}},$$

where Y_ijk_ = the phenotype measured on animal k, Temperature_i_ = effect of temperature group i (fixed effect; T_24-24-21_, T_19-19-19_, T_23-17-15_), and HCW_j_ = hot carcass weight j (regression coefficient); e_ij_∼NID(0, δ^2^e). Growth parameters A and B were analyzed with the same model (6), but without including the effect of HCW. Initially, the effect of room was also included in the model, but since this was not significant it was removed.

Data are presented as lsmeans ± s.e.m. Results are determined statistically significant with associated p levels of 0.05 or less, and determined a trend with associated p levels between 0.05 and 0.10. Because FCE and ShoulderW:HamW are ratios, values were log-transformed for analysis. Because Ham%, Loin%, and Shoulder% are based on proportions, after dividing by 100, values were transformed with the logit transformation for analysis. Least squares means of log and logit transformed values were then back-transformed and presented. Note that standard errors are not presented since they are based on the log and logit transformed values, however, transformed log and logit values with their s.e.m. can be found in Supplemental Tables S1 and S2. Subsequently, errors of all traits analyzed with models (5) and (6) were evaluated for normality by Q-Q plots and tested for normality with the Anderson-Darling test. Evaluation for normality showed that normality was rejected for the traits DBWG, RFI, and log-transformed values of FCE (model 5), and for B, HamW, LoinW, and logit-transformed values of Loin% (model 6). Since no clear outliers could be determined, no data was removed from the dataset to attempt to improve normality. Since linear regression models, such as models (5) and (6), are generally robust to violations of the normality assumption, since the number of observations in this study (158) is not too small, and since, for example, rank-based inverse normal (RIN) transformed values cannot be back-transformed, no further transformation was performed^18^. However, rank-based differences between temperature groups after RIN transformations of the traits DBWG, RFI, FCE (model 5), and B, HamW, LoinW, and Loin% (model 6) can be found as Supplementary Tables S1 and S2; after RIN transformation, normality assumption was met for all traits.

Because some traits failed the normality assumption, partial Spearman correlation coefficients between all traits were estimated for non-transformed values after adjusting first for the effect of temperature group; for the estimation of the correlation with feed efficiency, slaughter yields were also adjusted for the effect of HCW.

## Results

### Growth, feed intake and feed efficiency

**Figure 3:**
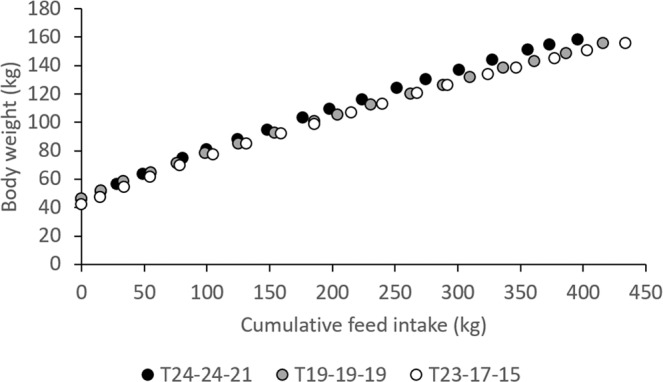
Body weight as a function of cumulative feed intake, in three temperature groups (T_24-24-21_, T_19-19-19_, T_23-17-15_).

Body weight as a function of feed intake is presented in Fig. 3 for each of the three temperature groups. Figure 3 suggests that T_24-24-21_ reaches slaughter weight on less feed. Equation 1 converged in 36 animals in T_24-24-21_, 27 animals in T_19-19-19_, and 29 animals in T_23-17-15_, i.e., in a total of 92 animals. In these animal, the curves fitted the data very well, as indicated by the goodness of fit, R^2^, of 90 to nearly 100%. The estimate of mature body weight, A, was significantly lower, and the estimate of the rate of maturation with respect to feed intake, B, was significantly higher in T_23-17-15_ than in T_24-24-21_ and T_19-19-19_ (Table 2). Equation 2 fitted the data nearly 100%. Mature feed intake was higher in T_23-17-15_ than in T_24-24-21_ (Table 2). Pigs with a higher B had a lower A (r = −0.92; P < 0.0001). Pigs with a higher B (r = −0.54; P < 0.0001) and lower A (r = 0.62; P < 0.0001) had a lower MFI.

**Table 2:** Average total (±s.e.m.), and least squares means (±s.e.m.) for each temperature group (T_24-24-21_,T_19-19-19_, and T_23-17-15_) of mature body weight (A), rate of maturation with respect to feed intake (B), mature feed intake (MFI), body weight one day before slaughter (BWsl), hot carcass weight (HCW), ham weight (HamW), shoulder weight (ShoulderW), loin weight (LoinW), proportion between shoulder weight and ham weight (ShoulderW:HamW), dressing percentage (Dressing%), and HamW, ShoulderW and LoinW as a percentage of HCW (Ham%, Shoulder%, and Loin%, respectively), analyzed with model 6.

Item	Total	T_24-24-21_	T_19-19-19_	T_23-17-15_
A (kg)	287 (±4.04)	293^x^ (±6.27)	295^x^ (±7.24)	271^y^ (±6.99)
B (per g)	1.57 (±0.0362)	1.57^x,y^ (±0.0551)	1.41^x^ (±0.0636)	1.72^y^ (±0.0614)
MFI (kg/d)	3.76 (±0.0377)	3.66^x^ (±0.0642)	3.72^x,y^ (±0.0624)	3.90^y^ (±0.0661)
BWsl (kg)	162 (±1.17)	158^x^ (±1.83)	170^y^ (±1.78)	156^x^ (±1.89)
HCW (kg)	128 (±0.905)	126^x^ (±1.42)	136^y^ (±1.39)	122^x^ (±1.45)
Dressing%	79.3 (±0.0981)	79.6^x^ (±0.157)	79.4^x^ (±0.169)	78.7^y^ (±0.170)
HamW (kg)	16.3 (±0.129)	16.1^x^ (±0.0816)	16.5^y^ (±0.0895)	16.2^x^ (±0.0882)
ShoulderW (kg)	10.6 (±0.0742)	10.5^x^ (±0.0683)	10.5^x^ (±0.0742)	10.9^y^ (±0.0739)
LoinW (kg)	2.70 (±0.0241)	2.82^x^ (±0.0318)	2.66^y^ (±0.0352)	2.59^y^ (±0.0348)
ShoulderW:HamW^a^	0.653	0.648^x^	0.639^x^	0.675^y^
Ham%^b^	12.7	12.6^x^	12.9^y^	12.7^x^
Shoulder%^b^	8.30	8.16^x^	8.23^x^	8.54^y^
Loin%^b^	2.10	2.20^x^	2.08^y^	2.02^y^
BFT (mm)	50.2 (±0.655)	49.0^x^ (±1.07)	51.0^x^ (±1.146)	50.6^x^ (±1.15)

^x,y^Values with different superscripts are significantly different (P < 0.05). ^a^Analysis was based on log-transformed data; values presented are backtransformed. ^b^Analysis was based on logit-transformed data; values presented are backtransformed.

**Figure 4:**
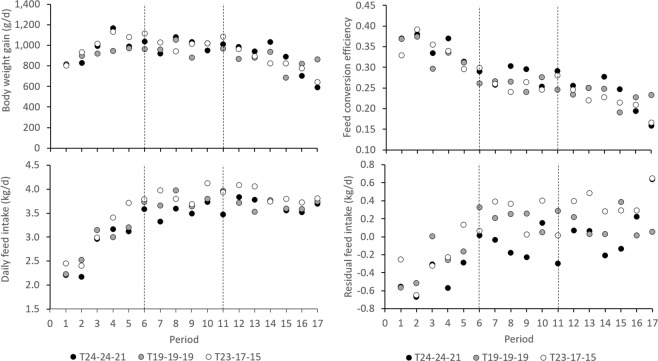
Average daily body weight gain (**a**), daily feed intake (**b**), feed conversion efficiency **(c**), and residual feed intake (**d**), in each of 17 periods of approximately one week each, spanning a total of 118–120 days, in each temperature group (T_24-24-21_, T_19-19-19_, and T_23-17-15_).

**Figure 5:**
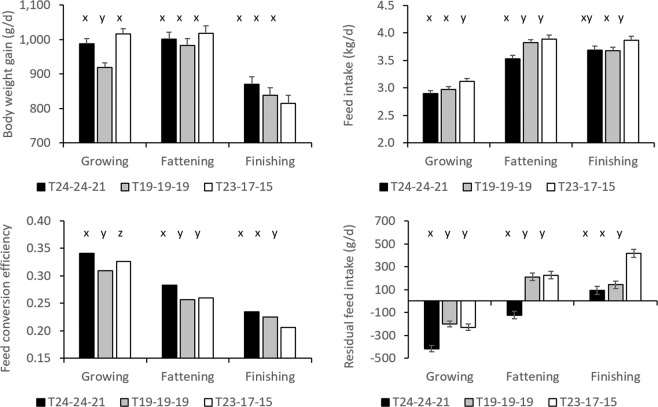
Average daily body weight gain (**a**), daily feed intake (**b**), feed conversion efficiency (**c**), and residual feed intake (**d**), in the growing, fattening, and finishing stages, in each temperature group (T_24-24-21_, T_19-19-19_, and T_23-17-15_). Within growth phase, bars with a different letter differ.

Daily body weight gain was highest during the fattening stage and lowest during the finishing stage (P < 0.0001; Fig. 4a). During the growing stage, T_19-19-19_ had lower DBWG than T_24-24-21_ (P = 0.0008) and T_23-17-15_ (P < 0.0001); DBWG did not significantly differ between the temperature groups during the fattening and finishing stages, but in the finishing stage, DBWG tended to be lower in T_23-17-15_ than in T_24-24-21_ (P = 0.0882) (Fig. 5a). DFI was lowest during the growing stage (P < 0.0001), and similar during the fattening and finishing stages (Fig. 4b). During the growing stage, animals in T_23-17-15_ had higher DFI than animals in T_24-24-21_ (P = 0.0021) and T_19-19-19_ (P = 0.0358); during the fattening stage, animals in T_24-24-21_ had lower DFI than animals in T_19-19-19_ (P = 0.0010) and T_23-17-15_ (P < 0.0001); during the finishing stage, animals in T_23-17-15_ had higher DFI than animals in T_19-19-19_ (P = 0.0494) and tended to have higher DFI than animals in T_24-24-21_ (P = 0.0754) (Fig. 5b).

Model (1) had an R^2^ of 66%; the contribution of BW^0.75^ (0.0315 ± 0.00794; P < 0.0001), DBWG (2.29 ± 0.202; P < 0.0001), and BFT (0.00683 ± 0.00208; P = 0.0013) were all significant, but the intercept (−0.0124 ± 0.244) was not significantly different from zero (P = 0.9596). Feed conversion efficiency was highest during the growing stage, and lowest during the finishing stage (P < 0.0001; Fig. 4c). Similarly, RFI was lowest during the growing stage, and highest during the finishing stage (P < 0.0001; Fig. 4d). During the growing stage, FCE was highest in T_24-24-21_ (P < 0.0001 with T_19-19-19_ and P = 0.0013 with T_23-17-15_) and lowest in T_19-19-19_ (P = 0.0002 with T_23-17-15_; Fig. 5c); RFI was lowest in T_24-24-21_ (P < 0.0001 with T_19-19-19_ and with T_23-17-15_) but similar between T_19-19-19_ and T_23-17-15_ (P = 0.4442; Fig. 5d). During the fattening stage, animals in T_24-24-21_ where more feed efficient than animals in T_19-19-19_ and T_23-17-15_ (P < 0.0001; Fig. 5c,d); during the finishing stage, animals in T_23-17-15_ where less feed efficient than animals in T_24-24-21_ and in T_19-19-19_ (P < 0.0001; Fig. 5c,d).

Animals with higher DBWG in the growing stage, also had a higher DBWG in the fattening stage (r = 0.19, P = 0.0153); pigs with higher DBWG in the fattening stage, had higher DBWG in the finishing stage (r = 0.43, P < 0.0001). Pigs with higher DFI in the growing stage had higher DFI in the fattening stage (r = 0.37, P < 0.0001), and pigs with higher DFI in the fattening stage, had higher DFI in the finishing stage (r = 0.56, P < 0.0001). Feed efficiency was positively correlated between all stages (r = 0.18 to 0.26 for FCE and r = 0.21 to 0.26 for RFI, P < 0.001). Animals with higher DBWG had higher DFI (r = 0.78, 0.81, and 0.85 during the growing, fattening, and finishing stages, respectively, P < 0.0001). As expected, animals with high FCE had low RFI (r = −0.90, −0.75, and –0.53 during the growing, fattening, and finishing stage, respectively, P < 0.0001). Since FCE is mostly determined by variation in DBWG, animals with high DBWG had higher FCE (r = 0.21 with P = 0.0094, and r = 0.50 and r = 0.69 with P < 0.0001 during the growing, fattening, and finishing stage, respectively). On the contrary, RFI is positively related with DFI (r = 0.48, 0.51, and 0.55 during the growing, fattening, and finishing stage, respectively, P < 0.0001), while the relationship between FCE and DFI was variable (r = −0.38 with P < 0.0001; r = −0.02 with P = 0.8217; and r = 0.26, P = 0.0012, during the growing, fattening, and finishing stage, respectively).

The relationship with growth curve parameters showed that animals with a faster maturation rate and those with lower mature body weights had significantly lower DBWG during the fattening (r = −0.31 with P = 0.0030, and r = 0.48 with P < 0.0001, respectively) and finishing stages (r = −0.70 and r = 0.79, P < 0.0001, respectively), and a lower DFI during the fattening (r = −0.18 with P = 0.0802, and r = 0.28 with P = 0.0073, respectively) and finishing stages (r = −0.68 and r = 0.72, P < 0.0001, respectively). This resulted in lower FCE during the finishing stage (r = −0.47 and r = 0.53, P < 0.0001, respectively).

### Hot carcass weight and slaughter yields

**Figure 6:**
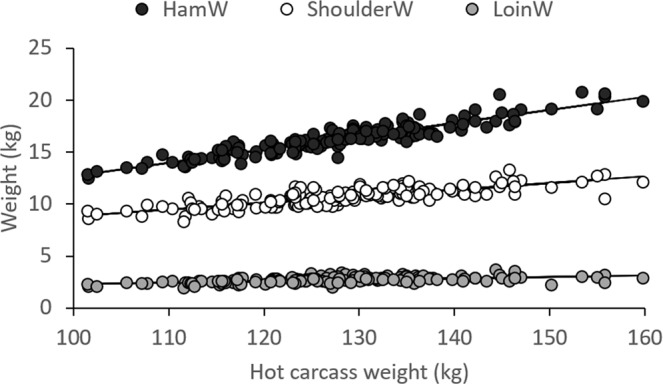
Ham weight (HamW), shoulder weight (ShoulderW) and loin weight (LoinW) as a function of hot carcass weight.

The regression coefficient of a linear regression between BWsl and HCW was 0.807 (±0.0115; R^2^ = 0.97), corresponding to a Dressing% of 81%. Since heavier carcasses were fatter (r = 0.29, P = 0.0003), Dressing% increased with HCW (r = 0.19, P = 0.0196). Heavier carcasses produced larger HamW, ShoulderW, and LoinW (r = 0.88, 0.81, and 0.55, respectively, P < 0.0001; Fig. 6). Regressing slaughter weights on HCW showed that for each kg of increase in HCW, HamW increased approximately 127 g, ShoulderW 64 g, and LoinW 14 g (P < 0.0001). However, since heavier carcasses were fatter and external fat is trimmed from the ham, shoulder, and loin before weighing, higher HCW resulted in a reduced Shoulder% (r = −0.20, P = 0.0120), Loin% (r = −0.34, P < 0.0001); and Ham% (r = −0.10), but the latter was not significant (P = 0.2189). Regressing slaughter yields on HCW showed that for each kg of increase in HCW, Shoulder% and Loin% reduced approximately 0.01 (P < 0.0001) and 0.005%, respectively (P = 0.0002), whereas Ham% did not significantly change.

Slaughter weight of animals depends on the management decision to send animals for slaughter at a certain (average batch) weight. This was at a higher BWsl in T_19-19-19_ than in T_24-24-21_ and T_23-17-15_. Consequently, animals in T_19-19-19_ had higher HCW than animals in T_24-24-21_ and T_23-17-15_ (Table 2). After adjustment for the effect of HCW, no significant differences existed between the temperature groups for BFT. HamW and Ham% were significantly higher in T_19-19-19_ than in T_24-24-21_ and T_23-17-15_, Dressing%, ShoulderW and Shoulder% were significantly higher in T_23-17-15_ than in T_24-24-21_ and T_19-19-19_, and LoinW and Loin% were significantly higher in T_24-24-21_ than in T_19-19-19_ and T_23-17-15_ (Table 2).

After adjustment for HCW and temperature group, pigs with higher DBWG in the growing stage tended to have leaner carcasses (r = −0.15, P = 0.0665), whereas BFT was higher in pigs with higher DBWG in the finishing (r = 0.17, P = 0.0389) and fattening stages (r = 0.23, P = 0.0041). Pigs with higher DFI during the fattening and finishing stages, and those with higher MFI had fatter carcasses (r = 0.19 with P = 0.0158, r = 0.24 with 0.0033, and r = 0.24 with P = 0.0030, respectively). In general, more feed efficient animals (i.e., with higher FCE or lower RFI) had higher ham and loin yields (Table 3). The relationship with growth curve parameters showed that faster maturing animals and those with lower mature body weights had significantly heavier HamW (r = 0.32 with P = 0.0022, and r = −0.23 with P = 0.0321, respectively) and higher Ham% (r = 0.32 with P = 0.0027, and r = −0.21 with P = 0.0458, respectively). In contrast, the relationships with ShoulderW and Shoulder% or LoinW and Loin% were r = 0.00 to 0.09. Faster maturing animals had lower BFT at slaughter (r = −0.22, P = 0.0384).

**Table 3:** Phenotypic correlations between feed conversion efficiency (FCE) and residual feed intake (RFI), with ham weight (HamW), shoulder weight (ShoulderW), loin weight (LoinW), the proportion between shoulder weight and ham weight (ShoulderW:HamW), and HamW (Ham%), ShoulderW (Shoulder%), and LoinW (Loin%) as a percentage of hot carcass weight.

Item	FCE^a^			RFI^a^		
	Growing	Fattening	Finishing	Growing	Fattening	Finishing
HamW^b^	0.24**	0.18*	0.13^†^	−0.22**	−0.15^†^	−0.19*
ShoulderW^b^	0.14^†^	0.10	0.14^†^	−0.09	−0.10	−0.25**
LoinW^b^	0.32***	0.17*	0.20*	−0.23**	−0.26**	−0.33***
ShoulderW:HamW^b^	−0.05	−0.02	0.05	0.09	−0.01	−0.09
Ham%^b^	0.24**	0.19*	0.14^†^	−0.23**	−0.16*	−0.18*
Shoulder%^b^	0.13	0.09	0.13	−0.08	−0.09	−0.26**
Loin%^b^	0.31***	0.17*	0.19*	−0.22**	−0.26**	−0.33***

^a^Adjusted for the effect of temperature group; ^b^adjusted for the effect of temperature group and hot carcass weight; ***P < 0.001; **P < 0.01; *P < 0.05; ^†^P < 0.10.

## Discussion

At nearly 30 million pigs in 2017, Spain has the largest pig population in the EU^19^. The swine sector is very efficiently structured and integrated, and has one of the lowest production costs^20^; Spain exported nearly 700 thousand tonnes of pork meat in 2016^21^. Spanish pig production is divided in the production of modern white pigs and (crossbred) Iberian pigs. The Iberian pig breed is the most important Mediterranean swine type (Sus mediterraneus), both in population size and in economic importance^22^. The breed is medium-sized, resistant to high summer temperatures, has a low prolificacy, a low basal metabolism, and early formation of fatty tissues, resulting in a high potential for fat accumulation, a low productivity, and a low feed efficiency^10,23^. Reduced performance, however, is amply compensated by its products that are destined to a niche market of highly priced dry-cured processed meat. According to Nieto *et al*.^10^, in Spain only 10% of the total amount of slaughtered Iberian pigs are 100% Iberian; the remaining 90% is crossed with Duroc. Crossbreeding Iberian sows with Duroc boars improves average daily gain, leanness, feed efficiency, carcass quality, and prolificacy, but reduces meat quality^24,25^. About 30% of purebred and crossbred Duroc × Iberian pigs are fattened in an extensive production system called ‘montanera’, where they exclusively feed on acorns, grass, herbs, roots, and bulbs, or are supplemented with concentrate feeding. The remaining 60%, all crossbred Duroc × Iberian pigs, are fed intensively on concentrate feed^10^. Primal cuts of the Iberian (crossbred) pig (ham or “jamon”, shoulder of “paleta”, and loin or “lomo”) are solely destined for the production of cured products. They are world-renown high-quality gourmet products that are protected by Spain’s Designation of Origin rules for food products.

### Growth curve parameters

Duroc × Iberian pigs that are produced intensively on concentrate feed are slaughtered at a minimum age of 10 months, at a life weight of about 140–150 kg; in our experiment, and in particular in T_19-19-19_, animals were slaughtered at life weight heavier than 150 kg. In many cases this slaughter weight is considerably higher than that of commercial white pig breeds, which may be slaughtered around 110–120 kg. Given that Duroc × Iberian pigs are small-sized pigs, in particular because of an absence of selection pressure in the Iberian breed, they are physiologically more mature than pigs from larger-sized commercial white breeds when compared at similar body weights. In addition, desire for lean meat, high growth rate, and high feed efficiency have resulted in commercial pigs that are slaughtered around peak lean growth rate, i.e., greatly below their mature size (generally at 50% or less^26^;). Physiologically, this corresponds to about the beginning of the fattening stage in the present study (Fig. 1). In contrast, subcutaneous, inter-, and intramuscular fat are important characteristics for the production of cured products^27^, therefore, (crossbred) Iberian pigs are finished far beyond optimum lean tissue growth rate, accepting a serious depression in feed efficiency (Fig. 5c,d).

Growth curves converged in 59% of the animals in the present study, which presented a unique dataset to investigate the influence of temperature variation on growth curve parameters. Mature mass, A, may be safe for protein mass, however, there is little evidence of a mature lipid mass^26^. Consequently, Taylor^28^ proposed defining asymptotic mass at a standard for body fat reserves where lipid constitutes a proportion of 0.15 of adult weight. Since Iberian pigs and their crosses are predisposed to a high lipid deposition, and lipid body mass was unknown in the present study, ‘asymptotic body weight at a constant lipid mass’ must have been overestimated despite convergence of growth curves in model (1). When fitting a time-dependent growth curve (such as the Gompertz or Brody curve), a negative correlation between mature weight and maturation rate with respect to time (age) has been commonly reported in several livestock species^29^. In the present study, pigs that matured faster with respect to feed intake (i.e., with higher B) also grew to a lower mature body weight in all temperature groups. As animals mature, they become less feed efficient^30^. Our results indicated that animals that matured faster and those that reach a lower mature weight had a slower DBWG, a lower DFI and lower FCE during the fattening and finishing stages, but had heavier hams constituting a larger percentage of carcass weight. The latter may partly result from the observation that the faster maturing animals had leaner carcasses.

Our results show that maturation rate, B, was highest in pigs that grew in the growing period in a warm environment (T_23-17-15_) and lowest in those that grew in a cold environment (T_19-19-19_). In addition, pigs in T_23-17-15_ also reached a significantly lower mature weight. The influence of temperature on mature body weight is relevant because, according to Taylor^28^, metabolic age, which is derived from mature body weight (calculated as chronological age or time variables divided by A^−0.27^), influences many important life events, including age at weaning, age at maturity, and total life span. Since animals in all temperature groups came from the same genetic lines it may be expected that they are estimated to grow to a similar mature weight. Therefore, the depression in estimated mature body weight may have resulted from colder temperatures in T_23-17-15_ during the fattening and finishing stages that resulted in a reduction in feed efficiency: this may have been compensated for if they would have finished in a more temperature-favorable environment.

### Daily body weight gain, daily feed intake, and feed efficiency

Thermal comfort zones are determined by the balance between external heat load, internal heat production, and heat dissipation. Beyond the lower and upper critical temperatures, animals resort to behavioral and physiological coping mechanisms to add *vs*. eliminate additional heat load or increase *vs*. reduce heat production. Older pigs have a broader thermoneutral zone and a lower upper critical temperature than younger pigs^31^. When temperatures decrease beyond the lower critical temperature of the thermal comfort zone, feed intake increases to support extra heat production^32^. In contrast, when temperatures increase beyond the upper critical temperature of the thermal comfort zone, a reduction in feed intake may reduce heat production in two ways. Firstly, metabolic rate resulting from the heat increment of feeding on internal heat production can be significantly reduced by reducing feed intake^33^. Secondly, heat production is further reduced when body weight gain reduces as a result of a reduced feed intake, or as a mechanism to further reduce metabolic rate^34^. Therefore, a profound depression in both feed intake and growth rate is a common observation in all heat-stressed livestock^35^.

Several studies showed that feed intakes drop at temperatures beyond 20 °C (e.g.^6,17,36^). Brown-Brandl *et al*.^37^ reported a maximum value of the thermal comfort zone ranging between 17.4 and 23.2 °C. In the present study, pigs were housed individually, whereas pigs generally grow-finish in group housing. Group housing may influence the range of the thermal comfort zone, in particular with respect to the lower critical temperature through the ability to huddling together^38^. Group housing is also known to influence feed intake, however, Renaudeau *et al*.^6^ observed at high ambient temperatures that the intake difference between pigs in individual vs. group housing did not appear to change with ambient temperature. The thermal comfort zone is furthermore influenced by genetic line. Genetic selection aimed at increasing the amount of lean tissue growth rate leads to a reduced capacity for coping with heat stress^6^. For example, Rauw *et al*.^17^ observed a negative correlation between DBWG in a thermoneutral environment with that during heat stress, indicating that faster growth resulted in lower robustness to heat stress, while pigs with higher DBWG during heat stress had a lower growth rate in more favorable temperatures. Although thermal comfort zones have not been established in Iberian pigs or their crosses, it is expected that slower growth in these lines and their crosses results in an increased upper critical temperature, therefore, Duroc x Iberian pigs are likely more robust to heat stress than pigs from commercial white breeds.

**Figure 7:**
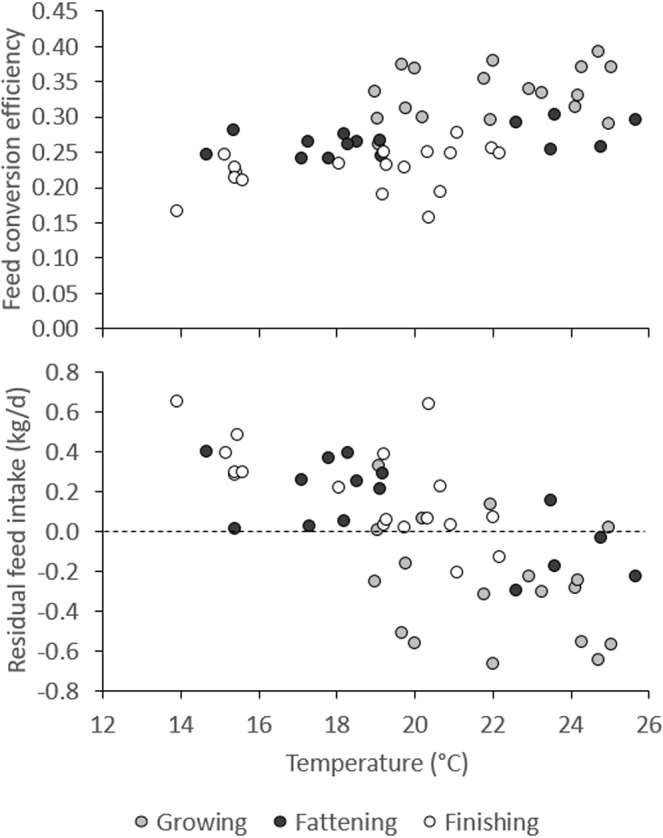
Feed conversion efficiency and residual feed intake as a function of temperature, for each of 17 periods of about a week in three temperature groups, during the growing, fattening, and finishing stages.

In the present study, T_23-17-15_ and T_24-24-21_ were the warmest environments in the growing stage, T_24-24-21_ was the warmest environment in the fattening stage, and T_23-17-15_ was the coldest environment in the finishing stage. Based on the thermal comfort zone estimated in white pigs, temperatures were high during the growing and fattening stage in T_24-24-21_ (summer months) and, given several high maximum scores, also during the growing stage in T_23-17-15_ (end of summer). However, during the growing stage, DFI was highest in T_23-17-15_, and similar between T_24-24-21_ and T_19-19-19_; during the fattening stage, DFI was indeed lowest in T_24-24-21_. In the growing stage, pigs had highest DBWG in the warmest environments (T_24-24-21_ and T_23-17-15_) and lowest in the coldest environment (T_19-19-19_); in the finishing stage, DBWG tended to be lower in the coldest environment (T_23-17-15_). Feed conversion efficiency (FCE) and residual feed intake (RFI) are both commonly used measures of feed efficiency. Since FCE is positively correlated with DBWG, whereas RFI is positively correlated with DFI, depending on the extent to which environmental temperature affects growth and feed intake, it may affect both measurements of feed efficiency differently^17^. Figure 3 shows that pigs growing-fattening under constantly warmer temperatures (T_24-24-21_) reached slaughter weight on the lowest amount of feed. Indeed, our results show that feed efficiency was generally highest in the warmer environments. To investigate this further, DBWG, DFI, and feed efficiency were plotted against average temperatures calculated in each of the three temperature groups in each of the 17 periods. Figure 7 shows an improvement of feed efficiency in warmer environments. When adjusted for the effect of growth stage and temperature group, Spearman correlations were r = 0.32 (P = 0.0215) with FCE and r = −0.50 (P = 0.0002) with RFI. Improved efficiency appeared to result from a reduction in DFI (r = −0.34, P = 0.0153), whereas animals were able to maintain DBWG (r = 0.13, P = 0.3767) when environmental temperatures increased. Regression analysis showed a significant depression in feed intake of approximately 49 (P = 0.0009) and 28 g per °C (P = 0.0315) in the fattening and finishing stages, respectively. Results were very similar when correlations were estimated with the THI index instead. Similarly, Rauw *et al*.^17^ observed a clear increase in feed efficiency in pigs during repeated exposure to heat stress, both measured as FCE and as RFI. In that experiment, during heat stress, all pigs from a commercial line and divergent lines selected for high and low RFI significantly reduced their feed intake, whereas DBWG only significantly reduced in the fast and lean growing commercial line. When animals lose weight, feed efficiency is only negatively affected when measured as FCE, as weight loss penalizes FCE but not RFI. This is because, when feed efficiency is measured as RFI, an animal that loses less BW (with a less negative value of DBWG) is still considered more feed efficient than an animal that loses more BW (with a more negative value for DBWG) than expected based on its, now reduced, feed intake. However, negative DBWG will always result in a negative FCE. The latter is also the reason why feed conversion ratio (DFI/DBWG) cannot be used as a measure of feed efficiency when individuals in a population lose weight^17^. Although an improvement in feed efficiency in pig production is considered positive, it is clear that any reduction in DBWG results in increased time to slaughter and increased fixed costs related to time on farm, and will thus negatively affect economic production efficiency. In addition, it clearly results in negative animal welfare^39^.

### Slaughter yield

Animals that were slaughtered at a higher BWsl had heavier carcasses. Since internal organ growth is proportional to body weight with a fractional power of less than unity, while fat tissue grows faster than muscle tissue, it is generally observed that Dressing% increases with carcass weight, and in particular in fattier types of pigs^40,41^. This was supported by the results of the present study. Increased weight, therefore, results in an increase in the percentage of fat but a decrease in the percentages of muscle, bone and skin. In the present study, increased carcass weights were related to reduced Shoulder% and Loin%, but not Ham%. This may be caused by a larger proportion of (untrimmed) fat accumulation in the ham than in the other primal cuts. Forero *et al*.^42^ observed a decrease in Ham% and Shoulder% of 0.5 and 0.39 for each 10 kg increase of HCW in purebred Iberian barrows. In their study, purebred Iberian pigs with an average HCW of 120 kg had a Ham%, Shoulder% and Loin% of 8.65, 5.79 and 1.49%, respectively. These values are lower than those in the present study; purebred Iberian pigs are fatter than their crosses. In contrast, the values by Landgraf *et al*.^41^ of Ham% and Shoulder% of 13.6 and 7.07%, respectively, in Piétrain × (Large White × Landrace × Leicoma) females and boars with an average HCW of 118 kg are more similar to those observed in the present study. The proportion between ShoulderW and HamW is of particular interest to Iberian pig production. Because of the difficulty to commercialize too small shoulders, there is an interest from the Iberian pig industry to increase the proportion of ShoulderW:HamW^42^. Forero *et al*.^42^ observed a quadratic relationship between ShoulderW:HamW and HCW in purebred Iberian pigs, in which the proportion increased until 134 kg HCW, and thereafter decreased. However, in our study, no significant relationship between ShoulderW:HamW and HCW was found.

Heat-induced alterations in the hierarchy of tissue accretion rates may result in altered carcass phenotypes^43^. For example, heat stress increases insulin concentrations which may explain why heat-stressed animals do not mobilize adipose tissue despite being in a hypercatabolic state, resulting in fatter carcasses^43^. Results of the present study show that, after correction for variation in carcass weight, no significant differences were found in BFT between the three temperature groups. This suggests that, in the present study, warm environments appeared not hot enough to result in fatter carcasses, and neither did the climate that was coldest during fattening (T_23-17-15_) result in body tissue mobilization or leaner carcasses. After adjustment for carcass weight, pigs in T_23-17-15_ had a significantly lower Dressing% than pigs in the other two environments. Since BFT was not significantly different between the three temperature groups, this may have resulted from the significantly higher DFI during the colder finishing stage, resulting in larger digestive tracts^44^. Our results indicate that T_24-24-21_ temperatures appeared to favor loin yield, T_19-19-19_ favored ham yield, and T_23-17-15_ favored shoulder yield. Chmielowiec-Korzeniowska *et al*.^45^ also observed a higher loin yield, but not ham or shoulder yield, in group-housed pigs fattened during the summer season (between approximately 25 to 18 °C) than in pigs fattened during the winter season (between approximately 13-18 °C). Why different parts are favored at different temperatures is not clear. In our study, warmer temperatures improved feed efficiency, in addition, improved feed efficiency improved ham and loin yields.

## Conclusion

Environmental temperature significantly influenced growth curve parameters and therefor the growth trajectory. Comparison between temperature groups indicated, for the temperatures and breed choice in the present experiment, a general tendency to higher growth rates and lower feed intakes in warmer temperatures. Linear regression of production traits as a function of temperature suggests that temperature in particular resulted in reduced feed intakes, whereas pigs were able to maintain body weight gains. As a consequence, warmer temperatures resulted in higher feed efficiency. Pigs with higher feed efficiency had higher ham and loin yields; different environmental temperatures favored different primal cuts. Although improvement in feed efficiency is desirable, heat stress should always be avoided since it may negatively affect animal health and welfare.

## Supplementary information

Supplementary information.

## Data Availability

The datasets generated during and/or analyzed during the current study are available from the corresponding author on reasonable request.
