# Supplementary material for: Impact of environmental temperature on production traits in pigs
Source: Sci Rep. 2020 Feb 7;10:2106. doi: 10.1038/s41598-020-58981-w (PMC7005870; doi:10.1038/s41598-020-58981-w)
Supplement: Supplementary file 1 — Supplementary information. [file 41598_2020_58981_MOESM1_ESM.pdf]

# Supplementary information

## Impact of environmental temperature on production traits in pigs

Wendy M. Rauw, Eduardo de Mercado de la Peña, Luis Gomez-Raya, Luis Alberto García Cortés, Juan José Ciruelos, & Emilio Gómez Izquierdo

**Supplementary Table S1** Average least squares means (± s.e.m.) for each temperature group ($T_{24-24-21}$, $T_{19-19-19}$, and $T_{23-17-15}$) of a) log-transformed values of the proportion between shoulder weight and ham weight (ShoulderW:HamW), and logit transformed values of ham weight (HamW), shoulder weight (ShoulderW) and loin weight (LoinW) as a percentage of HCW (Ham%, Shoulder%, and Loin%, respectively), and b) rank-based inverse normal transformed values of rate of maturation with respect to feed intake (B), HamW, LoinW, and Loin%, analyzed with model 6.

| a | $T_{24-24-21}$ | $T_{19-19-19}$ | $T_{23-17-15}$ |
|---|---|---|---|
| ShoulderW:HamW | -0.434$^{x}$ (± 0.00726) | -0.448$^{x}$ (± 0.00796) | -0.393$^{y}$ (± 0.00785) |
| Ham% | -1.94$^{x}$ (± 0.00568) | -1.91$^{y}$ (± 0.00623) | -1.93$^{x}$ (± 0.00614) |
| Shoulder% | -2.42$^{x}$ (± 0.00687) | -2.41$^{x}$ (± 0.00747) | -2.37$^{y}$ (± 0.00744) |
| Loin% | -3.80$^{x}$ (± 0.0121) | -3.85$^{y}$ (± 0.0134) | -3.88$^{y}$ (± 0.0132) |

| b | $T_{24-24-21}$ | $T_{19-19-19}$ | $T_{23-17-15}$ |
|---|---|---|---|
| B (per g) | 0.122$^{x}$ (± 0.155) | -0.501$^{y}$ (± 0.179) | 0.326$^{x}$ (± 0.176) |
| HamW (kg) | -0.103$^{x}$ (± 0.0519) | 0.115$^{y}$ (± 0.0570) | -0.0843$^{x}$ (± 0.0562) |
| LoinW (kg) | 0.427$^{x}$ (± 0.105) | -0.121$^{y}$ (± 0.116) | -0.403$^{y}$ (± 0.115) |
| Loin% | 0.528$^{x}$ (± 0.120) | -0.125$^{y}$ (± 0.133) | -0.442$^{y}$ (± 0.131) |

$^{x,y}$: Values with different superscripts are significantly different ($P < 0.05$).

**Supplementary Table S2** Average least squares means (± s.e.m.) for each temperature group ($T_{24-24-21}$, $T_{19-19-19}$, and $T_{23-17-15}$) of a) log-transformed values of feed conversion ratio (FCE), and b) rank-based inverse normal transformed values of daily body weight gain (DBWG), FCE, and residual feed intake (RFI), during the growing, fattening, and finishing stages, analyzed with model 5.

| a | $T_{24-24-21}$ | $T_{19-19-19}$ | $T_{23-17-15}$ |
|---|---|---|---|
| $FCE_{growing}$ | $-1.08^{x}$ (± 0.0142) | $-1.17^{x}$ (± 0.0138) | $-1.12^{y}$ (± 0.0147) |
| $FCE_{fattening}$ | $-1.26^{x}$ (± 0.0142) | $-1.36^{y}$ (± 0.0138) | $-1.35^{x}$ (± 0.0147) |
| $FCE_{finishing}$ | $-1.45^{x}$ (± 0.0142) | $-1.49^{x}$ (± 0.0138) | $-1.58^{y}$ (± 0.0147) |

| b | $T_{24-24-21}$ | $T_{19-19-19}$ | $T_{23-17-15}$ |
|---|---|---|---|
| $DBWG_{growing}$ | $0.157^{x}$ (± 0.133) | $-0.473^{y}$ (± 0.130) | $-0.379^{x}$ (± 0.139) |
| $DBWG_{fattening}$ | $0.0141^{x}$ (± 0.133) | $-0.154^{x}$ (± 0.130) | $-0.168^{x}$ (± 0.139) |
| $DBWG_{finishing}$ | $0.157^{x}$ (± 0.133) | $-0.0404^{x}$ (± 0.130) | $-0.115^{x}$ (± 0.139) |
| $FCE_{growing}$ | $0.600^{x}$ (± 0.122) | $-0.581^{y}$ (± 0.119) | $0.0470^{z}$ (± 0.127) |
| $FCE_{fattening}$ | $0.654^{x}$ (± 0.122) | $-0.411^{y}$ (± 0.119) | $-0.206^{y}$ (± 0. 127) |
| $FCE_{finishing}$ | $0.398^{x}$ (± 0.122) | $0.131^{x}$ (± 0.119) | $-0.549^{y}$ (± 0. 127) |
| $RFI_{growing}$ | $-0.671^{x}$ (± 0.118) | $0.385^{y}$ (± 0.115) | $0.258^{y}$ (± 0.122) |
| $RFI_{fattening}$ | $-0.828^{x}$ (± 0.118) | $0.398^{y}$ (± 0.115) | $0.412^{y}$ (± 0.122) |
| $RFI_{finishing}$ | $-0.397^{x}$ (± 0.118) | $-0.260^{x}$ (± 0.115) | $0.698^{y}$ (± 0.122) |

$^{x,y}$: Values with different superscripts are significantly different ($P < 0.05$).
